# High-Performance Mg_3_Sb_2-*x*_Bi*_x_* Thermoelectrics: Progress and Perspective

**DOI:** 10.34133/2020/1934848

**Published:** 2020-11-15

**Authors:** Airan Li, Chenguang Fu, Xinbing Zhao, Tiejun Zhu

**Affiliations:** ^1^State Key Laboratory of Silicon Materials, School of Materials Science and Engineering, Zhejiang University, 310027 Hangzhou, China; ^2^Max Planck Institute for Chemical Physics of Solids, Nöthnitzer Str. 40, 01187 Dresden, Germany

## Abstract

Since the first successful implementation of *n*-type doping, low-cost Mg_3_Sb_2-*x*_Bi*_x_* alloys have been rapidly developed as excellent thermoelectric materials in recent years. An average figure of merit *zT* above unity over the temperature range 300–700 K makes this new system become a promising alternative to the commercially used *n*-type Bi_2_Te_3-*x*_Se*_x_* alloys for either refrigeration or low-grade heat power generation near room temperature. In this review, with the structure-property-application relationship as the mainline, we first discuss how the crystallographic, electronic, and phononic structures lay the foundation of the high thermoelectric performance. Then, optimization strategies, including the physical aspects of band engineering with Sb/Bi alloying and carrier scattering mechanism with grain boundary modification and the chemical aspects of Mg defects and aliovalent doping, are extensively reviewed. Mainstream directions targeting the improvement of *zT* near room temperature are outlined. Finally, device applications and related engineering issues are discussed. We hope this review could help to promote the understanding and future developments of low-cost Mg_3_Sb_2-*x*_Bi*_x_* alloys for practical thermoelectric applications.

## 1. Introduction

Thermoelectric (TE) materials, which can convert heat into electric energy or vice versa without any moving parts, provide a promising solution to the current energy crisis [1]. In order to enable more practical applications in power generation and solid-state cooling, the key challenge is to improve the performance of TE materials, which is usually gauged by the dimensionless figure of merit, *zT*, *zT* = *S*^2^*σT*/(*κ*_L_ + *κ*_e_), where *S*, *σ*, *T*, *κ*_L_, and *κ*_e_ are the Seebeck coefficient, electrical conductivity, absolute temperature, and lattice and electronic components of thermal conductivity *κ*, respectively [2]. Aiming at obtaining a high *zT*, there are two primary directions in the TE research: one is to optimize the TE properties of established good materials mainly through the band and phonon engineering strategies [3, 4]; the other is to di*s*cover novel promising candidates based on the instruction of theoretical predictions or trial and error [5, 6].

With a constant endeavor of thermoelectricians in the past decades, more than ten semiconductor systems have been exploited with high *zT* above unity, some even higher than 2, e.g., V_2_VI_3_ compounds [7–10], IV-VI compounds [11–18], transition metal chalcogenides [19, 20], half-Heusler compounds [21–26], skutterudites [27–29], Zintl compounds [30–33], clathrates [34, 35], metal silicides [36–38], and Si_1-*x*_Ge*_x_* alloys [39, 40]. Among these good TE systems, most of them exhibit peak *zT* in the moderate-to-high temperature range (≥600 K), of which the practical applications would be power generation. In contrast, TE materials with high *zT* near room temperature (RT) are superior candidates for both low-grade heat power generation and solid-state cooling. Regarding cooling applications, there is a big market demand in a variety of industries, such as healthcare, automotive, semiconductors, and electronics. The global solid-state cooling market is estimated to be valued at USD 395 million in 2019 and is predicted to reach USD 641 million by 2024 [41]. Hence, the development of low-cost TE materials with high *zT* near RT is highly desirable.

Since the discovery in the mid-twentieth century [42], V_2_VI_3_ compounds [9, 43], including Bi_2_Te_3_, Sb_2_Te_3_, and Bi_2_Se_3_, and their solid solutions, have long been the best TE materials near RT, being the primary choice for the commercial cooling application. However, the relatively scarce element tellurium (see Table 1) is a potential impediment for the large-scale TE applications of V_2_VI_3_ compounds. In recent years, Mg-based semiconductors, e.g., *p*-type *α*-MgAgSb [44–46] and *n*-type Mg_3_Sb_2-*x*_Bi*_x_* [33, 47, 48], have been discovered with good TE performance near RT, comparable to V_2_VI_3_ compounds. Magnesium is the 8th most abundant element in the earth's crust [49]. TE materials containing low-cost magnesium could thus have a better prospect for large-scale applications.

Historically, the exploration of Mg-based TE materials began with Mg_2_Si-based compounds in the 1960s [50]. By alloying with Sn or Ge, the resulted band convergence and point defect scattering contribute to a peak *zT* above unity in Mg_2_Si_1-*x*_Sn*_x_* and Mg_2_Ge_1-*x*_Sn*_x_* solid solutions [36, 37, 51, 52]. A series of work has also been conducted to optimize their TE performance, including introducing dislocations and point defects [53–55]. In 2012, the TE properties of another Mg-based compound *α*-MgAgSb, which belongs to the Nowotny-Juza family [56], were reported by Kirkham et al. [44]. The synthesized *α*-MgAgSb sample with unignorable impurity phases still exhibits a potential *zT* of 0.56 at around 400 K. Later, by improving phase purity, a peak *zT* above unity was obtained in *p*-type MgAgSb samples by several groups [45, 46, 57, 58]. The hierarchical chemical bonding in *α*-MgAgSb leads to strong anharmonicity and intrinsically low sound velocity [59], which are responsible for the low thermal conductivity *κ* and high *zT* near RT [45, 46, 57]. However, MgAgSb has a complex phase transition process when cooling from its liquid state with two phase transition points at around 360°C and 300°C, respectively [60]. Additionally, the binary impurity phase, for instance, Ag_3_Sb, is also easily formed during the synthesis. These make it difficult to synthesize the high-purity *α*-MgAgSb (room temperature phase); particularly, the single crystal is not yet reported. Moreover, the high TE performance in *α*-MgAgSb is only obtained when doping it as *p*-type, and there is still no experimental report on successful electron doping.

**Figure 1:**
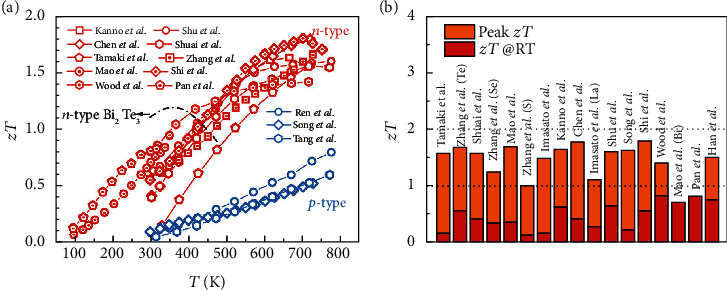
(a) Temperature-dependent *zT* for both *n*-type and *p*-type Mg_3_Sb_2-*x*_Bi*_x_*-based TE materials. The data are taken from Refs. [33, 48, 70–73, 77–83]. As a comparison, the *zT* of *n*-type Bi_2_Te_3-*x*_Se*_x_* is also shown [8]. (b) The peak and room temperature *zT* for *n*-type Mg_3_Sb_2-*x*_Bi*_x_*. The data are taken from Refs. [33, 48, 70–73, 80, 82, 84–89].

Intermetallic compound Mg_3_*X*_2_ (*X* = Sb, Bi) was first reported by Zintl and Husemann in the 1930s [61], later being classified as a member of Zintl phase compounds [62]. The electrical transport behavior of Mg_3_*X*_2_ was studied by Pincherle and Radcliffe in the 1950s [63]. Mg_3_Sb_2_ exhibited a *p*-type semiconducting behavior with an energy gap (*E*_g_) of 0.8 eV while Mg_3_Bi_2_ showed metallic behavior. The study on the TE properties of Mg_3_*X*_2_ began in the 2000s [64]. Despite the low *κ* and inexpensive raw elements, its *p*-type semiconducting behavior and inferior TE performance do not make it a good TE material [65–69]. In 2016, Mg_3_Sb_2-*x*_Bi*_x_* compounds were discovered to be a new promising *n*-type TE system by exhibiting a peak *zT* of 1.5 at around 700 K [70–73], more than twice higher than that of the *p*-type counterparts (Figure 1(a)). This exciting result also makes *n*-type Mg_3_Sb_2-*x*_Bi*_x_* standing out of the Zintl phase TE family, of which the family members are generally exhibiting good *p*-type TE properties, even rarely been electron-doped [74]. The successful realization of *n*-type Mg_3_Sb_2-*x*_Bi*_x_* results from the understanding of the Mg defect chemistry, and the Bi alloying promoted electron doping by reducing the bandgap [70, 71]. That is, only in the Mg-rich environment, for instance, adding slightly excess Mg in the raw experimental design, can *n*-type Mg_3_Sb_2-*x*_Bi*_x_* be obtained. Actually, this excess Mg supportive electron doping has been recognized in synthesizing *n*-type Mg_2_Si_1-*x*_Sn*_x_* TE materials previously, where excess Mg was thought to compensate the loss of Mg during the synthesis, and additionally, some excess Mg atoms could enter into the interstitial sites facilitating electron doping [54, 75, 76]. The role of excess Mg in Mg_3_Sb_2-*x*_Bi*_x_* is mainly suppressing the Mg vacancies while Mg interstitial is difficult to form, which will be discussed in detail in the later section.

Despite exhibiting a high *zT* of 1.5 at around 700 K, the power generators made from *n*-type Mg_3_Sb_2-*x*_Bi*_x_* might be challenging owing to the decomposition of the compounds and possible deterioration of electrical properties at elevated temperatures [90, 91]. Fortunately, even near RT, *n*-type Mg_3_Sb_2-*x*_Bi*_x_* also shows good *zT* of around 0.8 (Figure 1(b)), making it a promising alternative to the state-of-the-art *n*-type Bi_2_Te_3-*x*_Se*_x_* for solid-state cooling application. Although only 4 years have passed since the discovery of *n*-type Mg_3_Sb_2-*x*_Bi*_x_*, there are already many important advances achieved, including the improvement of TE performance [33, 48, 70–73, 78–80, 82, 92, 93], the understanding of the origin for good power factor [70, 71] and intrinsically low *κ* [94], the revelation of the carrier scattering mechanism near RT [82, 95, 96], the increasing awareness of Mg defect chemistry [70, 97, 98], and even the successful attempt of TE module [33].

The advances in *n*-type Mg_3_Sb_2-*x*_Bi*_x_* demonstrate a good paradigm of how a new TE material can be rapidly developed and even transferred into the lab-scale module verification by the current TE community. In the past two years, there have been two timely review articles discussing the recent progress of Mg_3_Sb_2_ and its derivatives: one is focusing on the design principle with a combination of theory and experiment [99] and the other on the manipulation of defects and electronic transport properties [100]. Readers who are interested in more details may refer to these two reviews. In this review, we focus on recent progress and perspective of the TE performance enhancement near RT. Started with the understanding of basic information of Mg_3_Sb_2-*x*_Bi*_x_*, the bonding and crystal structure, electronic structure, and phonon dispersion are discussed to reveal the intrinsic foundation of the transport properties. Then, several important aspects, including band engineering with Sb/Bi alloying, carrier scattering mechanism near room temperature, Mg defect chemistry, and extrinsic doping which affect the TE performance near RT, are summarized. Finally, we discuss the thermal stability of this system, which is a key step towards practical applications. We hope this review could help to promote the development of Mg_3_Sb_2-*x*_Bi*_x_* for future TE applications near RT.

## 2. Chemical Bonding and Structure

### 2.1. Basic Information about the Constituent Elements

The structure-property relationship is essential to understand and design advanced materials for practical applications. Here, before the comprehensive discussions of the structure-property relationship in the Mg_3_Sb_2-*x*_Bi*_x_* system, we first make a list of the basic physical and chemical properties of the elements Mg, Sb, Bi, Te, and Se (Table 1), making up the two near-RT TE systems, Mg_3_Sb_2-*x*_Bi*_x_* and Bi_2_Te_3-*x*_Se*_x_*. Several important points, which will be frequently referred to in the following sections, are first highlighted here. (i) The density of Mg is much smaller than that of Sb, Bi, Te, and Se. This could be an advantage of lightweight for *n*-type Mg_3_Sb_2-*x*_Bi*_x_* used for TE modules, compared to commercial Bi_2_Te_3-*x*_Se*_x_*. (ii) The melting point of Mg is the highest one among the listed elements. However, its lower boiling point, compared to Sb and Bi, suggests a higher vapor pressure and a potentially larger loss of Mg for Mg_3_Sb_2-*x*_Bi*_x_* at elevated temperatures [103]. (iii) The Pauling electronegativity (EN) of Mg is only 1.31, much smaller compared to O (3.44), suggesting the high reactivity of Mg metal. In the atmosphere, the surface of Mg metal is soon coated with a thin layer of oxide that partly inhibits reactivity, the so-called passivation. The passivation and high vapor pressure of Mg could be reasons why the actual compositions of the synthesized Mg_3_Sb_2-*x*_Bi*_x_* compounds are generally Mg-deficient if being nominally designed. (iv) The electronic configuration of Mg is 3*s*^2^, and hence, Mg usually has a +2 oxidation state in the ambient environment. The Pauling ionic radius of Mg^2+^ is 0.65 Å, which is much smaller than that of the Sb and Bi anions (their Pauling ionic radii are not available but should be larger than their atomic radii, by referring to the difference in the atomic and ionic radii of Te, Se, and O). This diminutive radius of Mg^2+^, even comparable to that of Li^+^ (0.6 Å), was thought to be one of the structural reasons leading to weak interlayer bonding and thus intrinsically low *κ* in Mg_3_Sb_2_ and Mg_3_Bi_2_ [94]. Additionally, this is perhaps also why Mg_3_Bi_2_ has also attracted attention as candidates for superionic conductor [104, 105] and Mg-ion battery [106, 107]. Furthermore, although having not been thoroughly studied, this diminutive radius of Mg^2+^ might also relate to the thermal instability of this system when working at elevated temperatures. (v) The difference in Pauling EN for the constituent elements of Mg_3_Sb_2_ is 0.74 (0.71 for Mg_3_Bi_2_), which is much larger that of Bi_2_Te_3_ (0.08) and the other good thermoelectrics, such as Sb_2_Te_3_ (0.05), PbTe (0.23), PbSe (0.22), GeTe (0.09), SnTe (0.14), SnSe (0.59), SiGe (0.11), Mg_2_Si (0.59), and ZnSb (0.4). This is an indication that the chemical bond of Mg_3_Sb_2_ and Mg_3_Bi_2_ has a larger component of ionic character, compared to above mentioned good thermoelectrics.

### 2.2. Crystal Structure

Mg_3_Sb_2_ and Mg_3_Bi_2_ crystallize in a trigonal anti-*α*-La_2_O_3_ type structure (space group *P*$\bar{3}$*m*1) below 1203 K and 976 K, respectively [108]. A cubic Mn_2_O_3_-type structure (space group *Ia*$\bar{3}$) was reported in some literature for Mg_3_*X*_2_ above the transition temperatures [109]. For Mg_3_Bi_2_, a neutron powder diffraction was carried out to determine the high-temperature phase by Barnes et al. [104]. A phase transition from the *α* phase (low temperature) to the *β* phase was observed at *T* = 730 ± 10°C. The *β*-Mg_3_Bi_2_ was determined to be a body-centered cubic structure. However, to our best survey of the literature, there is still no convincing structural information of *β*-Mg_3_Sb_2_. We tried to quench Mg_3_Sb_2_ and Mg_3_Bi_2_ samples into liquid nitrogen from temperature above 1203 K and 976 K, respectively. However, with the XRD measurement, the quenched crystals still show a trigonal structure without a trace of cubic structure, suggesting the phase transition might be fairly quick.

**Figure 2:**
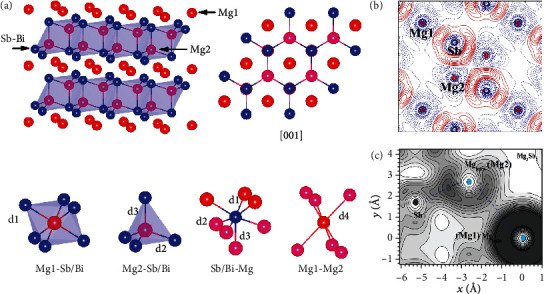
(a) Crystal structure of Mg_3_*X*_2_, the right figure is viewed from [001] direction. Drawn using *VESTA* [125]. The coordination of Mg1, Mg2, and *X* is presented below the crystal structure. (b) Static deformation electron density map on (110) plane of Mg_3_Sb_2_. (c) The partial charge densities for Mg_3_Sb_2_. Panel (b) is reproduced with permission from Ref. [121]. CC-BY-4.0. Panel (c) is reproduced with permission from Ref. [116]. Copyright 2019 Wiley Periodicals, Inc.

The crystal structure of trigonal Mg_3_*X*_2_ and the corresponding lattice parameters and bond lengths are shown in Figure 2(a) and Table 2, respectively. Under the Zintl concept, Mg_3_*X*_2_ can also be classified into the CaAl_2_Si_2_-type structure by rewriting it as MgMg_2_*X*_2_ [94]. Mg atoms take two different sites in the lattice: one is at the octahedral site surrounded by six *X* atoms, named as the Mg1 atom, and the other is at the tetrahedral site surrounded by four *X* atoms, named as the Mg2 atom. The Mg1 atom shows the most electropositive character and provides 2 electrons to the covalent bounded Mg_2_*X*_2_^2-^ network. This configuration not only provides a rough diagram to understand the structure-property relationship of Mg_3_*X*_2_ but also gives instruction about the site preference when alloying with other elements [113]. The Mg1 sites are favorably occupied when alloying with more electropositive alkaline earth metals and lanthanides, like Ca, Sr, Ba, La, and Yb [114–118], whereas Mg2 sites with more electronegative Zn, Mn, and Cd metals [119, 120].

Recently, Zhang et al. [99] performed a quantitative chemical bonding analysis of Mg_3_Sb_2_, from which they found that Mg1 and Mg2 atoms show close bonding character with the Sb atom with the atomic charge of +1.51 and +1.47, respectively (Figure 2(b)). The interlayer interaction in Mg_3_Sb_2_ is largely ionic with partial covalent nature, which is comparable to the intralayer interaction with the same type of bond [121]. These calculations give a rational explanation of the nearly isotropic thermal properties of Mg_3_*X*_2_ along the *ab*-plane and *c*-axis. These nearly isotropic thermal properties make Mg_3_*X*_2_ different from the other CaAl_2_Si_2_-type Zintl compounds, such as CaZn_2_Sb_2_ and SrZn_2_Sb_2_, showing anisotropic thermal properties [121]. With these findings, Zhang et al. argued the breakdown of the Zintl formalism in the *A*Mg_2_*X*_2_ system (*A* is an alkaline earth or a divalent rare earth element) [99], arousing the debate as to whether Mg_3_Sb_2_ is a Zintl phase [121, 122]. To address the debate, it is necessary to recall the definition of a Zintl phase. *AB*_2_*X*_2_ compounds with CaAl_2_Si_2_-type structure were defined, by Hoffmann et al. [113, 123], as Zintl phases since the structure can be described as the covalently bonded [*B*_2_*X*_2_]^*δ*−^ networks receiving electrons donated by the ionic *A*^*δ*+^ cations. In these compounds, the clear coexistence of the ionic cation and covalent anionic network is therefore the key feature of the Zintl phase and widely applied Zintl concept. Moreover, Kauzlarich et al. have also given a comprehensive discussion and historical review on the definition of Zintl phases [124], which can be referred to as the intermetallic compounds with mixed chemical bonding character of ionic and covalent bonds, generally composed of electropositive metal (alkali metal, alkaline earth element, and lanthanide) and posttransition metal or metalloid (i.e., from groups 13, 14, 15, or 16). From this point of review, it is not appropriate to consider Mg_3_Sb_2_ as Zintl phases as both the cationic and anionic parts are nearly ionic [99] although they are charge-balanced and the electron-counting rule is still applicable. It should be noted that the definition of the Zintl phase and Zintl concept discussed here is, in the narrow sense, the definition made by Hoffmann et al. [113, 123] and Kauzlarich et al. [124].

In addition to the Mg-Sb interactions, the calculations by Sun et al. [116] show that there exists a bonding interaction between the Mg1 and Mg2 atoms, in the conduction band minimum (CBM) region (Figure 2(c)). Since the distance between Mg1 and Mg2 is fairly large (Table 2) and also their close atomic charge [121], this interaction between 3*s* levels of Mg1 and Mg2 would be much weaker than that of Mg-Sb bonds. In short, compared to the typical CaAl_2_Si_2_-type Zintl compounds, the chemical bonding of Mg_3_*X*_2_ shows a nearly isotropic character in the Mg-Sb bonds between the interlayer and intralayer and an additional Mg1-Mg2 interaction at the CBM. This unique bonding character in Mg_3_*X*_2_, partly originating from the similar chemical environment of Mg1 and Mg2 atoms, is crucial to understand their electronic and phononic structures and thus the electrical and thermal transport properties.

### 2.3. Electronic Structure

**Figure 3:**
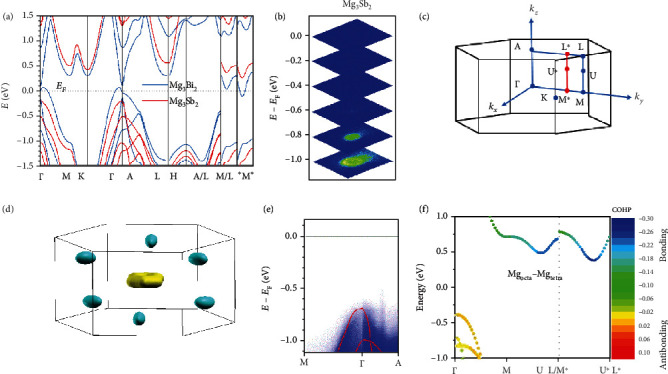
(a) Band structure of Mg_3_Sb_2_ and Mg_3_Bi_2_. (b) A series of constant energy maps of Mg_3_Sb_2_ by ARPES. (c) Brillouin zone (BZ) and high-symmetry points in BZ of the trigonal structure of Mg_3_*X*_2_. (d) The diagram of Fermi surfaces of Mg_3_Bi_2_. (e) ARPES spectra along the high-symmetry directions of Mg_3_Sb_2_, the red line is the corresponding calculated band structures. (f) The band-resolved projected Crystal Orbital Hamiltonian Population (COHP) for the interaction between Mg1 and Mg2 in Mg_3_Sb_2_. Panels (a, b, d, e) are reproduced with permission from Ref. [48]. Published by The Royal Society of Chemistry. Panel (f) is reproduced with permission from Ref. [116]. Copyright 2019 Wiley Periodicals, Inc.

We now move to understand the electronic structure of Mg_3_*X*_2_. The calculated band structures of Mg_3_Sb_2_ and Mg_3_Bi_2_ are juxtaposed in Figure 3(a). Mg_3_Sb_2_ is an indirect semiconductor with the calculated bandgap *E*_g_ ranging from 0.4 to 0.7 eV based on different calculation methods [70, 71, 110, 116, 126, 127], while Mg_3_Bi_2_ is predicted to be a topological nodal line semimetal with the coexistence of electron and hole pockets near Fermi level *E*_F_ [128–130]. Experimentally, an *E*_g_ of about 0.18 eV was estimated from the resistivity below 390 K for undoped Mg_3_Sb_2_ [131], while a much larger value of about 0.8 eV was reported by estimating the gap of the liquid phase of Mg_3_Sb_2_ [132]. A similar *E*_g_ of 0.8 eV was also reported in the 1950s [63]. The Fourier transform infrared spectroscopy measurement was performed to estimate the optical gap of Mg_3_Sb_2-*x*_Bi*_x_* solid solutions, yielding a similar value of about 0.28 eV for Mg_3_Sb_1.5_Bi_0.5_ [80] and Mg_3_SbBi [92]. Additionally, a direct revelation of the *E*_g_ for a semiconductor is possible by carrying out the angle-resolved photoemission spectroscopy (ARPES) on its *n*-type single crystals [26]. Pan et al. recently reported the ARPES study on *n*-type Mg_3_Sb_2_ single crystal, from which a forbidden gap is clearly observed (Figure 3(b)) and an *E*_g_ > 0.6eV is estimated for Mg_3_Sb_2_ [48]. But since the conduction band was not observed, further study is necessary to accurately determine the real *E*_g_. The ARPES study was also performed for Mg_3_Bi_2_ single crystal, and its semimetal feature was confirmed [129].

From Figure 3(a), it is easily observed that the valence band maximum (VBM) of both Mg_3_Sb_2_ and Mg_3_Bi_2_ is near the *Γ* point, the center of the first Brillouin zone (Figure 3(c)). In contrast, the conduction band minimum (CBM) of Mg_3_*X*_2_ locates at the U^∗^ point in the M^∗^L^∗^ direction, away from the high-symmetry points. To simultaneously demonstrate the shape and valley number of VBM and CBM, the Fermi surfaces of Mg_3_Bi_2_ are provided (Figure 3(d)). There are two distinct features in the VBM and CBM of Mg_3_Bi_2_. First, there are six electron pockets but only one hole pocket, suggesting a high band degeneracy *N*_v_ of 6 in CBM and a low *N*_v_ of 1 in VBM. High *N*_v_ is usually thought to be beneficial for high TE performance since the *zT* is proportional to the expression *μN*_v_*m*_b_^∗3/2^/*κ*_L_ [11, 21], where *μ* is the carrier mobility and *m*_b_^∗^ is the single-band effective mass. The difference in band degeneracy of CBM and VBM might explain why Mg_3_(Sb,Bi)_2_ shows better *n*-type TE performance [70, 71], as shown in Figure 1(a).

Another distinct feature is that the six electron pockets are nearly spherical while the hole pocket shows an obvious anisotropy along the *k*_*x*_‐*k*_*y*_ plane and *k*_*z*_ direction. This anisotropy in VBM is well embodied in the calculated effective masses along the two directions [71, 133, 134]. For example, in the calculation by Zhang et al., *m*_*xx*_^∗^(*m*_*yy*_^∗^) was estimated to be 1.15*m*_e_ and *m*_*zz*_^∗^ is 0.15*m*_e_ [71]. It is worth mentioning that the band anisotropy has recently been experimentally confirmed via the ARPES study on the Mg_3_Sb_2_ single crystal (Figure 3(e)), giving different effective masses of 0.9*m*_e_ and 0.16*m*_e_ along the two directions, respectively [48]. Moreover, a single crystal study on Mg_3_Bi_2_ had also confirmed this valence band anisotropy by measuring the electrical resistivity *ρ* along the *ab*-plane and *c*-axis, where the *ab*-plane was found to show a twice larger *ρ* [135]. The *ρ* along the *ab*-plane and *c*-axis was also reported for the Mg_3_Sb_2_ single crystals. However, since the studied single crystals were undoped ones and the very high *ρ* indicates that the Fermi level still lies in the forbidden gap, the band anisotropy on the VBM of Mg_3_Sb_2_ cannot be concluded [135]. The hole-doped Mg_3_Sb_2_ single crystals are required to observe the anisotropy in electrical transport along the *ab*-plane and *c*-axis.

Regarding the high *N*_v_ in the CBM of Mg_3_*X*_2_, another interesting question arises as to why the CBM locates off the high-symmetry points. This is an important question awaiting answers. If CBM locates at the U point, the electron pockets will be shared by the second Brillouin zone, and thus, the band degeneracy in the CBM will halve. One possible answer is traced to the electron structure calculation by Sun et al. [116], where they reported on a bonding interaction between the Mg1 and Mg2 atoms in the CBM region of Mg_3_Sb_2_, which is particularly stronger in the U^∗^ point than the other positions (Figure 3(f)). Even further, Han et al. recently found that this Mg1-Mg2 interaction in Mg_3_Sb_2_ can be even weakened by replacing Sb with Bi to increase the Mg1-Mg2 distance, leading to a more dispersive CBM although its position is still at the U^∗^ point [89]. Therefore, the unique Mg1-Mg2 interaction in Mg_3_*X*_2_ might be related to the origin of the CBM position, but further studies, both theoretically and experimentally, are still necessary before drawing a convincing conclusion. An experimental study of the CBM of Mg_3_*X*_2_ and the solid solution Mg_3_Sb_0.75_Bi_1.25_ by ARPES was attempted by Pan et al. [48]. Although their single crystals are all electron-doped with the carrier concentration *n* in the magnitude of 10^19^ cm^−3^, there is still no obvious electron pocket observed in the first Brillouin zone by ARPES. This indicates that higher electron-doped single crystals with *n* over 10^20^ cm^−3^ are necessary to experimentally reveal the CBM by ARPES.

### 2.4. Phononic Band Structure

**Figure 4:**
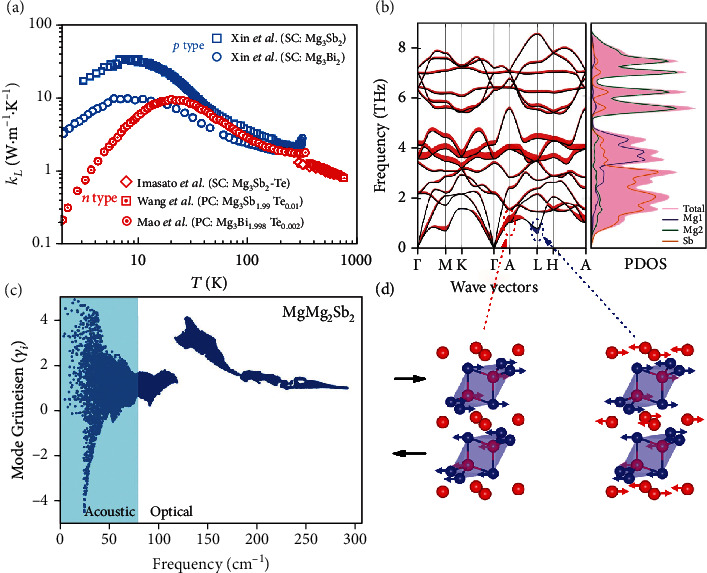
(a) The experimental *κ*_L_ of Mg_3_*X*_2_ and their alloys, where SC and PC denote single-crystalline and polycrystalline samples, respectively. The data are taken from Refs. [33, 96, 135, 142]. (b) Phonon dispersion and partial density of states (PDOS) of Mg_3_Sb_2_. (c) Mode Grüneisen parameter of Mg_3_Sb_2_. (d) The diagram of atomic displacement corresponding to the transverse at the A point (large positive *γ*_*i*_) and at the L point (large negative *γ*_*i*_). Panel (b) is reproduced with permission from Ref. [99]. CC-BY-4.0. Panels (c, d) are reproduced with permission from Ref. [94]. Copyright 2018 Elsevier Ltd.

Besides the high *N*_v_, the other key reason leading to the high TE performance of *n*-type Mg_3_Sb_2-*x*_Bi*_x_* is their low *κ*_L_.  Figure 4(a) presents the experimental *κ*_L_ for both single-crystalline and polycrystalline Mg_3_*X*_2_ and their solid solutions. In most experimental reports, the *κ*_L_ of Mg_3_*X*_2_ at room temperature is smaller than 2 Wm^−1^ K^−1^, which is comparable to that of Bi_2_Te_3_. This “anomalously” low *κ*_L_ of Mg_3_*X*_2_ is to some extent unexpected, considering its simple crystal structure and the light element Mg. In general, compounds with a complex crystal structure, i.e., a large number of atoms in the unit cell, often have intrinsically low *κ*_L_, because the considerable optical phonons in complex structures suppress the frequency of the acoustic phonons in the phase space and result in low group velocity of acoustic modes [136]. Compounds with heavy elements can also have low *κ*_L_ due to the low sound velocity of the acoustic phonons [137]. However, Mg_3_*X*_2_ has a simple crystal structure with only five atoms in the unit cell, and thus, the origin of its low *κ*_L_ becomes a very important question. The phononic band structure of pure Mg_3_Sb_2_ was reported by Peng et al. [94]. As shown in Figure 4(b), the thickness of the lines with colors represents the degree of anharmonicity. For the low-frequency acoustic branch, Mg_3_Sb_2_ shows a large degree of anharmonicity. If only taking the phonon-phonon Umklapp scattering into account, *κ*_L_ is proportional to the expression $A\bar{M}\theta_{\text{D}}\delta^{3}/\left(\gamma^{2}N^{2/3}T\right)$, where *A* is a collection of physical constant, $\bar{M}$ the average mass of atoms of a crystal, *θ*_D_ the Debye temperature, *δ*^3^ the volume per atom, *N* the number of atoms in the primitive unit cell, and *γ* the Grüneisen parameter reflecting the anharmonicity of the lattice [138]. The calculated *γ* for Mg_3_Sb_2_ is 1.83, which is higher than that of CaMg_2_Bi_2_ (*γ* = 1.48) and CaMg_2_Sb_2_ (*γ* = 1.44) having the same crystal structure [94]. The strong anharmonicity had also been confirmed by studying the *κ*_L_ of Mg_3_*X*_2_ using the Debye-Callaway model [135]. Comparatively, the other good TE materials with intrinsically low *κ*_L_ also show a large *γ*, such as PbTe (1.45) [139], AgSbTe_2_ (2.05) [138], BiCuSeO (1.5) [140], MgAgSb (1.93) [59], and SnSe (3.13) [141].

The calculated mode Grüneisen parameter of Mg_3_Sb_2_ by Peng et al. [94] is presented in Figure 4(c). It is found that the phonons of Mg_3_Sb_2_ with frequency below 1 THz show both large positive and negative mode Grüneisen parameters, reflected by the red shadow at A point and blue shadow at L point, respectively. Both A and L points involve the shear movement of atoms in the *ab*-plane, as shown in Figure 4(d). More specifically, the same atoms in different layers move towards totally inverse direction at both A and L points, which could be one of the reasons for large shear stress between layers at these points [94]. Except for the large anharmonicity, Mg_3_Sb_2_ also shows a low sound velocity of 2587 m·s^−1^, while CaMg_2_Sb_2_ has an average sound velocity of 3317 m·s^−1^ [94], which also contributes to their low *κ*_L_. Compared to Mg_3_Sb_2_, there is an obvious softening in the phonon density of states of Mg_3_Bi_2_ [143], owing to the heavier element Bi. This is why the *κ*_L_ of Mg_3_Bi_2_ is smaller than that of Mg_3_Sb_2_ [135].

Going further, there is still an interesting question concerning the thermal properties of Mg_3_*X*_2_. That is, why the large anharmonicity occurs in Mg_3_*X*_2_ but not in isoelectronic substituted CaMg_2_*X*_2_. Peng et al. [94] argued that the undersized Mg ions do not obey the octahedral bonding rules, leading to the instability of the octahedral site. Zhang et al. [99] thought that the higher formation enthalpy of Mg_3_*X*_2_ might lead to their relative instability and thus the soft mode and low *κ*_L_. Because there are few experimental works focusing on the understanding of the phononic structure of Mg_3_*X*_2_ at present, future studies, for example, using inelastic phonon scattering [144, 145], might help to reveal the origin of large anharmonicity in Mg_3_*X*_2_.

The anisotropy of *κ*_L_ is also a worthy question requiring a detailed discussion. In the original work of Tamaki et al. [70], the sound velocities of longitudinal acoustic mode along the *ab*-plane and *c*-axis are calculated to be 4160 m·s^−1^ and 4730 m·s^−1^, respectively, from which they concluded a nearly isotropic character in thermal conduction of Mg_3_Sb_2_. This conclusion is further supported by the chemical bonding analysis by Zhang et al. [121] where they found that Mg_3_Sb_2_ exhibits a nearly isotropic three-dimensional bonding network with the interlayer and intralayer bonds being mostly ionic and similar. The calculated *κ*_L_ along the *ab*-plane and *c*-axis is very close. Experimentally, Song et al. [146] prepared the texture-enhanced Mg_3_Sb_2-*x*_Bi*_x_* samples, from which they found that the lattice thermal conductivities are almost the same along the pressing direction and in-plane one. In short, the advantage of the nearly isotropic thermal and electrical conductions in *n*-type Mg_3_Sb_2-*x*_Bi*_x_* is that one does not need to specially take care of the direction of polycrystalline samples when doing the high-temperature thermal and electrical properties. This is actually not the case for the other layered TE materials, for example, Bi_2_Te_3_-based compounds, for which the *zT* can be overestimated if not measuring all the transport properties in the same direction [10, 147, 148].

## 3. Optimization of TE Properties

The chemical bonding, crystal, electronic, and phononic structures, discussed in the above section, lay the foundation for Mg_3_*X*_2_ as good thermoelectric materials. In practical experiments, the optimization of carrier concentration, engineering of the band and phononic structures, and regulation of electron and phonon scattering mechanisms are effective strategies to achieve high TE performance. Forming Mg_3_Sb_2-*x*_Bi*_x_* solid solutions is the most prevailing means to achieve a high *zT* (Figure 1). This is because Bi alloying could generate multiple beneficial effects, synergistically regulating both the electrical and thermal transport properties. Mg vacancy defects, which can directly affect the type of carriers, together with extrinsic chemical doping, are crucial for tuning the electrical transport properties of Mg_3_*X*_2_. Moreover, the electron scattering mechanism is another knob for achieving high electrical conductivity and good TE performance near RT. In this section, we focus on the discussions of Sb/Bi alloying, Mg vacancy defects, chemical doping, and carrier scattering mechanism, from which we will try to point out the ways towards high TE performance near RT.

### 3.1. Sb/Bi Alloying

Isoelectronic alloying to form solid solutions is a widely used strategy in TE research, of which the main purpose is to suppress the *κ*_L_ by introducing strong point defect scattering of phonons [149–151]. One key issue underlying the usage of this strategy is whether the solid solution can be formed in a full composition range (or whether there is a miscibility gap). The miscibility gap occurs in several TE systems, such as Mg_2_Si_1-*x*_Sn*_x_* [152–154], Ti_1-*x*_Zr*_x_*NiSn [155, 156], and Ti_1-*x*_Zr*_x_*CoSb [157, 158]. Luckily, there seems no miscibility gap in the Mg_3_Sb_2-*x*_Bi*_x_* system [33, 47, 73]. Intuitively, this can be understood partly from the mismatch in the lattice parameter of pristine compounds. For Mg_3_Sb_2_ and Mg_3_Bi_2_, the differences in the lattice parameter of both *a* and *c* directions are less than 2.5% (Table 1), while in the Mg_2_Si_1-*x*_Sn*_x_* system, the difference is larger than 6% [159]. This relatively smaller mismatch in the lattice parameter could to some extent explain the almost unaffected electron mobility of *n*-type Mg_3_Sb_2-*x*_Bi*_x_*, which will be discussed in detail later together with the change of electronic structure.

**Figure 5:**
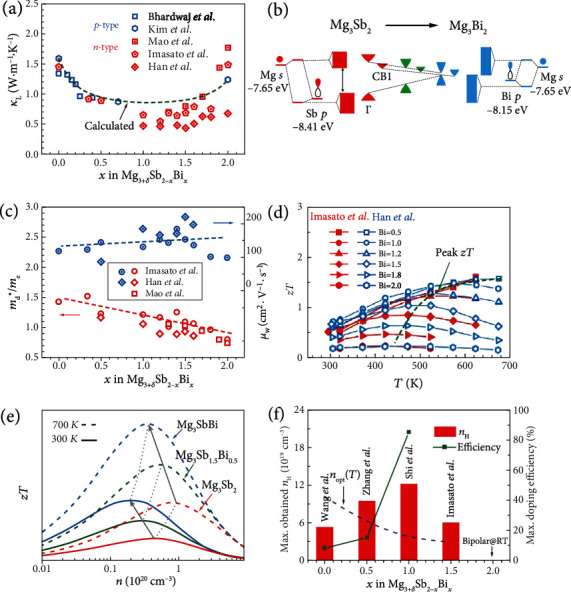
(a) *κ*_L_ at RT versus Bi alloying content for the Mg_3+*δ*_Sb_2-*x*_Bi*_x_* system. The data are taken from Refs. [33, 47, 67, 68, 89]; the dashed line is modeled only considering the mass fluctuation-induced point defect scattering of phonons. (b) Schematic illustration of the hybridization in Mg_3_*X*_2_ and the evolution of band structure, inspired by the work of Zhang et al. and Pan et al. [48, 99]. These energies of valence atomic orbitals are taken from the Periodic Table of Atomic Orbital Energies [172]. (c) The density of states (DOS) effective mass and weighted mobility versus Bi content. The data are taken from Refs. [33, 47, 89]. (d) Temperature dependence of *zT* for the Mg_3+*δ*_Sb_2-*x*_Bi*_x_* system with different Bi contents. The data are taken from Refs. [47, 89]. (e) Schematic carrier concentration dependence of *zT* for Mg_3_Sb_2-*x*_Bi*_x_* using the SPB model [164, 165]. (f) The experimentally obtained maximum Hall carrier concentration and the corresponding doping efficiency versus Bi content for the Mg_3+*δ*_Sb_2-*x*_Bi*_x_* system. The purple dashed line indicates the estimated *n*_opt_ at 700 K obtained from Figure 5(e). The experimental data are taken from Refs. [47, 73, 118, 173].

Similar to the other TE solid solution systems, such as PbTe_1-*x*_Se*_x_* [160], Zr_1-*x*_Hf*_x_*NiSn [150], and Zr_1-*x*_Hf*_x_*CoSb [159], the experimental *κ*_L_ for the Mg_3_Sb_2-*x*_Bi*_x_* system also presents a “U-shape” curve (Figure 5(a)), suggesting the efficacy in suppressing the lattice thermal transport with the enhanced point defect scattering of phonons. Near the pristine Mg_3_Sb_2_ and Mg_3_Bi_2_ sides, Bi/Sb alloying leads to a quick decay of *κ*_L_, while in the region with 0.4 ≤ *x* ≤ 1.6, the *κ*_L_ does not change obviously. Since the radius difference between Sb and Bi is relatively small, we tried to calculate the *κ*_L_ of the solid solution by only considering the mass fluctuation-induced point defect scattering of phonons [149]. As shown in Figure 5(a), the calculated line matches relatively well with the experimental results near the Mg_3_Sb_2_ side, indicating that the phonon scattering mainly comes from the mass fluctuation. Besides the effect of alloying on lattice thermal conductivity *κ*_L_, the change of sound velocity, probably originating from the alloying-induced alternation of phonon dispersion, could also influence *κ*_L_ [161]. When alloying with Mg_3_Bi_2_, the sound velocity will decrease gradually from 2587 m/s of Mg_3_Sb_2_ to 2055 m/s of Mg_3_Bi_2_ [94, 143], indicating lattice softening and probably lower *κ*_L_. This may be one of the reasons why the experimental results deviate from the calculated line in Figure 5(a). Additionally, there is a problem that needs to be paid attention to when comparing the *κ*_L_ and *zT* from different groups. Since Mg_3_Sb_2-*x*_Bi*_x_* alloy has been widely studied, the choices of heat capacity, either from the measurement or using the Dulong-Petit value, could to some extent influence the estimated *κ*_L_ and *zT*. Regarding this point, an empirical heat capacity equation was summarized by Agne et al. by including the Dulong-Petit value and temperature-dependent effects based on theory and experiment, which can be used without running *C*_*p*_ experiments on each sample: *C*_*p*_[J · g^−1^ · K^−1^] = 3*NR*/*M*_w_(1 + 1.3 × 10^−4^*T* − 4 × 10^3^*T*^−2^) (for temperatures 200K ≤ *T* ≤ 800K), where 3*NR* = 124.71J · mol^−1^ · K^−1^, *M*_w_ is the molecular weight of the formula unit being considered, and *T* is the absolute temperature [143]. To directly use this formula or take it as a standard reference for future studies is recommended so that a relatively fair comparison in the *κ*_L_ and *zT* can be reached.

In addition to the suppression of *κ*_L_, there is also a tremendous change in the electronic structure of the Mg_3_Sb_2-*x*_Bi*_x_* system when changing *x* from 0 to 2 since Mg_3_Sb_2_ and Mg_3_Bi_2_ are semiconductor and semimetal, respectively (Figure 3(a)). This offers the opportunity to modulate the band structure of the Mg_3_Sb_2-*x*_Bi*_x_* solid solutions enabling desirable TE performance at different temperatures. Before going into the performance modulation, we first try to qualitatively understand the band structure evolution from Mg_3_Sb_2_ to Mg_3_Bi_2_ based on the molecular orbital analysis (Figure 5(b)). The valence electrons of Mg and Sb elements are 3*s* and 5*s*5*p*, respectively, mainly contributing to the formation of chemical bonding. The energy level of the Mg 3*s* orbital is higher than that of the Sb 5*p* orbital. When the Mg 3*s* and Sb 5*p* orbitals come together to form the two molecular orbitals, the upper antibonding state is mainly composed of the Mg 3*s* orbital while the lower bonding state mainly with Sb 5*p*. This real-space analysis of bonding formation can directly reflect the composition of the band structure in reciprocal space. As shown from the projected DOS analysis [127, 162, 163], the CBM is mainly contributed by the Mg *s* orbital while the Sb *p* orbital dominates the VBM. When the Sb atom is substituted by the Bi atom, it will lead to two main changes. On the one hand, the energy difference between the Mg 3*s* and Bi 6*p* orbitals is smaller than that between the Mg 3*s* and Sb 5*p* orbitals, leading to the reduced *E*_g_ in Mg_3_Bi_2_. On the other hand, the Bi 6*p* orbital is more dispersive than Sb 5*p*, which can further reduce the *E*_g_ and also result in lighter conduction and valence bands. These qualitative analyses suggest that both *E*_g_ and conduction band effective mass will become smaller in the band evolution from Mg_3_Sb_2_ to Mg_3_Bi_2_.

Experimentally, the change in the density of state effective mass *m*_d_^∗^, *m*_d_^∗^ = *N*_v_^3/2^*m*_b_^∗^, which was derived from the measured *S* and *n*_H_ using the single parabolic band (SPB) model [164, 165], is presented in Figure 5(c), which indeed decreases with increasing Bi content. Moreover, the *m*_b_^∗^ obtained from the calculated electronic structure of the Mg_3_Sb_2-*x*_Bi*_x_* system was also reported by Han et al. [89], exhibiting the same trend. Beneficial from the reduced *m*_b_^∗^, the weighted mobility *μ*_w_ = *μ*_0_(*m*_d_^∗^/*m*_e_)^3/2^ increases, where *μ*_0_ is the mobility of a carrier at *k*_B_*T* higher than the band edge [166, 167]. Weighted mobility is also influenced by the scattering process when alloying with Bi. However, the alloying scattering is not so strong to decrease the carrier mobility. The reasons for this could be twofold: (i) the conduction band is dominated by the 3*s* states of Mg, and thus, the anion substitution does not significantly affect the conduction band transport, (ii) similar to what happens in the half-Heusler TE solid solutions [24, 159], where the small radius difference in the alloying and host atoms owing to lanthanide contraction does not induce a strong alloy scattering potential responsible for the negligible change in the carrier mobility. Hence, besides the suppressed *κ*_L_, the weak alloy scattering of carriers and the reduced *m*_b_^∗^ are additional advantages for Mg_3_Sb_2-*x*_Bi*_x_* solid solutions to achieve better TE performance.

Going further, we now discuss the effect of the reduced *E*_g_ on the TE properties of Mg_3_Sb_2-*x*_Bi*_x_*. In most of the previous TE studies, relatively large *E*_g_, which can suppress the thermal excitation of minority carriers [3, 168], is generally desirable for obtaining a high peak *zT* at elevated temperatures. Since the possible TE applications of Mg_3_Sb_2-*x*_Bi*_x_* solid solutions are near RT, a too-large *E*_g_ is not really necessary as long as it can still effectively suppress the bipolar effect near RT. The optimum *E*_g_ of a TE material given by Sofo and Mahan is around 6–10 *k*_B_*T* [169], corresponding to 0.15–0.26 eV at 300 K. Figure 5(d) shows the temperature-dependent *zT* for *n*-type Mg_3_Sb_2-*x*_Bi*_x_* with different Bi contents [47, 89]. It is obvious to see that the temperature at which the peak *zT* occurs decreases with increasing Bi content. This gives an additional advantage for practical experiments. Namely, the optimal carrier concentration *n*_opt_ will become smaller (Figure 5(e)) and thus easier to be achieved experimentally [168, 170].

In short, Bi alloying in Mg_3_Sb_2_ has exhibited multiple beneficial effects on the TE properties. First, it can significantly suppress the phonon transport but weakly affect the carrier transport. Second, the lightened *m*_b_^∗^ contributes to higher *μ*_w_. Third, the reduced *E*_g_ makes the *n*_opt_ easy to be achieved experimentally. Moreover, there is a large room to tune the peak *zT* of Mg_3_Sb_2-*x*_Bi*_x_* for applications at different temperatures. If aiming at power generation application, Sb-rich Mg_3_Sb_2-*x*_Bi*_x_* alloys have the advantage to achieve a high peak *zT* at elevated temperatures [70–73, 92]. However, owing to the solubility and efficiency of dopants, in the Sb-rich Mg_3_Sb_2-*x*_Bi*_x_* alloys, the experimentally maximum carrier concentration might still be lower than the *n*_opt_, particularly for Mg_3_Sb_2_ (Figure 5(f)). Therefore, more efficient dopants are waiting to be found [171]. In contrast, to get high TE performance near RT, Bi-rich Mg_3_Sb_2-*x*_Bi*_x_* alloys with smaller *E*_g_ are enough, in which the smaller *n*_opt_ is easier to be reached, as recently demonstrated by Imasato et al. [47], Mao et al. [33], and Pan et al. [48].

### 3.2. Carrier Scattering Mechanism

**Figure 6:**
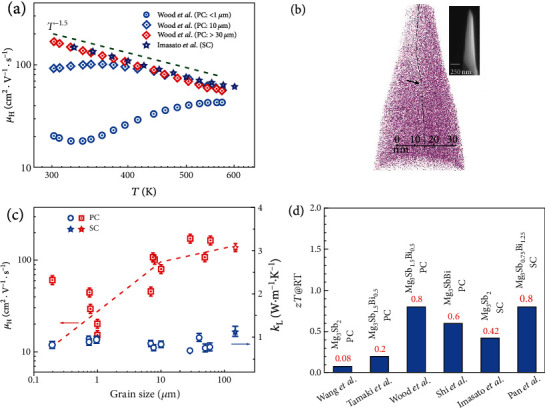
(a) Temperature-dependent *μ*_H_ for Mg_3_Sb_2-*x*_Bi*_x_* synthesized with different grain sizes. The data are taken from Refs. [78, 82]. (b) 3D reconstruction of the atom distribution of the microtip of *n*-type Mg_3_Sb_2_. The upper-right inset is a scanning electron microscope image of the APT specimen. (c) The *μ*_H_ and *κ*_L_ at RT versus grain size for Mg_3_(Sb,Bi)_2_ solid solutions, adapted from Ref. [48] with the data taken from Refs. [33, 47, 48, 78, 79, 166, 178]. (d) *zT* at RT for SC and PC samples with different compositions and preparing methods. The data are taken from Refs. [48, 70, 73, 82, 96, 136]. Panel (b) is reproduced with permission from Ref. [98]. Copyright 2019 Wiley-VCH.

The carrier scattering mechanism is a very important aspect to understand the electrical transport of TE materials, which helps to find suitable ways to improve TE performance by enhancing the carrier mobility and electrical conductivity. Since the discovery of *n*-type Mg_3_Sb_2-*x*_Bi*_x_* alloys [70, 71], an “anomalous” increase trend of *μ*(*T*) and *σ*(*T*) near RT is frequently observed in the doped samples with small grain sizes (Figure 6(a)), which was initially explained as ionized impurity scattering [70, 72, 79, 85]. Ionized impurity scattering-dominated carrier transport is commonly observed in the lightly doped semiconductors near and below RT [174], which is quickly weakened with increasing carrier concentration that induces a strong screen of the ionized impurity centers [175]. That is why in most good TE materials (typically heavily doped semiconductors), the *μ*(*T*) and *σ*(*T*) above RT generally decrease monotonously due to the dominated acoustic phonon scattering [176].

Back to *n*-type Mg_3_Sb_2-*x*_Bi*_x_* alloys, Kuo et al. [95] carefully analyzed the initially published experimental results and argued that the change from the ionized impurity scattering to acoustic phonon scattering near RT happens too fast to be satisfactorily explained by the conventional models using Matthiessen's rule. By considering the grain boundary region as an effectively separate phase rather than a scattering center, they put forward a two-phase model, which could successfully reproduce the experimentally observed *σ*(*T*). Moreover, they predicted a large improvement in the room temperature *zT* if the grain boundary resistance is eliminated. Later, using atom probe tomography, the grain boundary phase is identified to be Mg-deficient (approximately 5 at%) (Figure 6(b)), which is attributed to be responsible for the high grain boundary resistance [98]. Aiming at improving the *μ* and *σ* near RT by suppressing the grain boundary resistance, one direct way is to simply reduce the number of grain boundaries to get coarse-grained polycrystalline samples, which can be achieved by either increasing the sintering temperature [78, 82] or post-annealing under Mg atmosphere if initial grain sizes are small [82]. Furthermore, melting followed by a sintering process has recently been reported as an effective way to prepare samples with large grain sizes [73, 92, 177]. Some experimental results also indicate that Mg_3_Sb_2-*x*_Bi*_x_* samples with high Bi content can have large grain sizes under the same preparing condition [47], which is probably due to their low melting points.

Beyond the coarse-grained polycrystalline samples, the prediction of Kuo et al. [95] directly points out that the single-crystalline Mg_3_Sb_2-*x*_Bi*_x_* samples could have the ceiling *μ* and *σ* near RT due to the almost-completely eliminated grain boundary resistance. In experiments, Imasato et al. [96] reported on the TE properties of *n*-type Te-doped Mg_3_Sb_2_ single crystals, synthesized by the Sb-flux method [135] and Mg-vapor annealing [82], of which the *σ* indeed exhibits a metallic behavior with a typical *T*^−1.5^ dependence (Figure 6(a)), indicating the acoustic phonon scattering-dominated carrier transport and the absence of ionized impurity scattering [96]. Later, Pan et al. [48] reported on a direct growth of *n*-type Y-doped Mg_3_Sb_0.75_Bi_1.25_ single crystals by the Mg-flux method, which exhibits a high *zT* of ~0.82 at 315 K that is comparable to the state-of-the-art *n*-type room temperature Bi_2_Te_3-*x*_Se*_x_*-based TE alloys [8, 10, 147]. This series of work demonstrates a good research relay towards high-performance near-RT Mg_3_Sb_2-*x*_Bi*_x_* thermoelectrics based on the accurate understanding of the carrier scattering mechanism.

As extended discussions, we think there are another two points relating to this grain boundary resistance that are worth noting. On the one hand, the *μ*_H_ of polycrystalline samples at RT tends to have a slower increase when the grain size reaches above ~10 *μ*m, while the *κ*_L_ only changes slightly with grain size (Figure 6(c)). Kuo et al. recently pointed out that there will be an overestimation of *κ*_L_ for small grain polycrystalline samples because of high electrical resistance at the grain boundaries [179]. All of these indicate coarse-grained polycrystalline samples of tens of *μ*m are preferred to have a similar TE performance to the single crystals with the same composition. As summarized in Figure 6(d), the coarse-grained samples and the single crystals indeed exhibit a close *zT* of approximately 0.8 at RT for Mg_3_Sb_2-*x*_Bi*_x_* solid solutions [48, 135]. Since the growth of centimeter-sized *n*-type single crystals is still challenging, coarse-grained polycrystalline samples could currently be preferable choice for practical applications.

On the other hand, it is the grain boundary phase which has a high resistance that leads to the “anomalous” *μ*(*T*) and *σ*(*T*) near RT. Ideally, the grain boundary is thought of as a kind of very thin interface between two grains in a polycrystalline material. However, in real materials, the grain boundary region could be more complicated than a thin interface due to the segregation of second phases or the change of composition [180, 181]. Good TE materials could particularly be the case since they always need to be alloyed or doped to tune the electrical and thermal properties for high performance, which unavoidably leads to complicated microstructures [8, 182, 183]. Hence, a deep microstructure analysis to know the grain boundary region better with advanced techniques is highly necessary [184, 185]. For *n*-type Mg_3_Sb_2_ polycrystalline samples, the grain boundary phase is found to be a 10 nm region with Mg deficiency [98], resulting in the high electrical resistance. One possible indication thereby is if there are ways to make the grain boundary phase have an equal or even lower resistance than the matrix, the “anomalous” *μ*(*T*) and *σ*(*T*) near RT might vanish. This might explain why in some experiments, even with the same preparation method, using highly metallic dopants or codoping, like Sc, Y, and some other transition metals, could to some extent tune the “anomalous” *μ*(*T*) and *σ*(*T*) near RT [72, 85, 93]. The underlying reason might be that these highly metallic cation dopants make the grain boundary region have lower resistance compared to the less metallic anion dopants Te, Se, and S.

### 3.3. The Excess Mg and Chemical Doping

Intrinsic point defects, including vacancy, interstitial atoms, and antisite defects, could have important effects on both the electrical and thermal transport properties of TE materials [9, 186] and hence were studied extensively, such as Bi_Te_^−^ antisite defect in Bi_2_Te_3_ [9, 187, 188], interstitial Ni in ZrNiSn [26, 189, 190], and Ag vacancy in MgAgSb [58, 191]. Mg vacancy and interstitial Mg are two commonly observed point defects in Mg_2_(Si,Ge,Sn)-based TE materials [54, 55, 76], which could serve as acceptor and donor, respectively. For Mg_2_Ge and Mg_2_Sn, the type of carrier depends on the chemical environment of synthesis. A Mg-excess environment will lead to *n*-type transport behavior, while Mg deficiency to *p*-type [76]. For Mg_2_Si, interstitial Mg is the dominant point defect in both Mg-excess and Mg-deficient environments, and hence, it always shows an *n*-type transport behavior [76].

**Figure 7:**
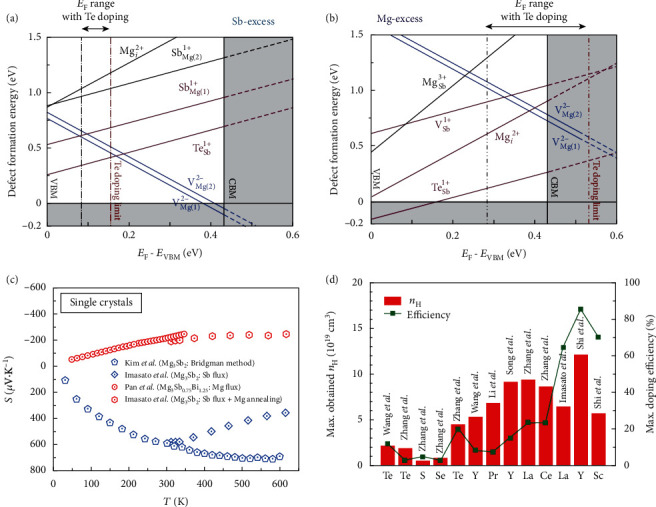
Defect formation energy in Mg_3_Sb_2_ in (a) Sb-excess and (b) Mg-excess conditions, respectively. (c) The Seebeck coefficient of Mg_3_Sb_2_ and Mg_3_(Sb,Bi)_2_ single crystals, grown under different chemical conditions. The data are taken from Refs. [48, 68, 96]. (d) The experimentally obtained maximum *n*_H_ in Mg_3_(Sb,Bi)_2_ samples and the calculated doping efficiency for different dopants. The blue color columns represent dopants at anion sites and the red at cation sites. The doping efficiency is calculated using the measured *n*_H_ divided by the theoretical one obtained assuming each doping atom offers one electron. The data are taken from Refs. [71, 73, 84, 86, 88, 92, 115, 118, 136, 173, 192, 193]. Panels (a, b) are reproduced with permission from Ref. [97]. Copyright 2018 Elsevier Ltd.

Similarly, the Mg_3_Sb_2-*x*_Bi*_x_* system is also vitally affected by the chemical environment of synthesis [48, 70, 97]. To explore the dominant defect type, first-principles calculations are commonly used to give a possible prediction [70, 97]. As shown in Figure 7(a), in the Sb-excess environment, Mg vacancy is the most stable defect near CBM. The *n*-doping by replacing Sb with Te fails to work since its formation energy is higher than that of Mg vacancy. Therefore, Te doping cannot shift the *E*_F_ into CBM if the chemical environment of synthesis is Sb-excess. In contrast, in the Mg-excess environment (Figure 7(b)), the formation energy of Mg vacancy is significantly increased, enabling the success of *n*-doping by substituting Te on the Sb site. Moreover, interstitial Mg has higher formation energy in both Sb-excess and Mg-excess conditions, indicating that only excess Mg in the synthesis process cannot help to shift the*E*_F_into CBM.

Experimentally, the transport properties of single crystals grown from the liquid flux methods (Sb-excess and Mg-excess) match well the prediction of the defect calculations, as summarized in Figure 7(c). Mg_3_Sb_2_ single crystals synthesized by using the vertical Bridgman method [68] and Sb-flux method [135] show the transport behavior of an intrinsic semiconductor with a large positive Seebeck coefficient. Te-doped Mg_3_Sb_2_ single crystals had also been successfully grown from the Sb-flux method, which however still shows the positive Seebeck coefficient, indicating Te doping itself cannot shift the *E*_F_ into CBM if the crystals are Mg-deficient [96]. Imasato et al. did a post-annealing of those Te-doped Mg_3_Sb_2_ single crystals under Mg-vapor, which successfully changed the transport behavior of the single crystals from *p*-type to *n*-type [96]. Very recently, using Mg as the flux, Pan et al. first reported a direct growth of Y-doped *n*-type Mg_3_Sb_2_ and Mg_3_Sb_0.75_Bi_1.25_ single crystals [48]. Additionally, they found that the undoped Mg_3_Sb_2_ single crystal shows a weak *p*-type transport behavior despite grown out from the Mg-flux [48]. This suggests that interstitial Mg is difficult to enter into the lattice of Mg_3_Sb_2_, in agreement with the calculated phase diagram by Ohno et al. [97], where they found that the solubility of both excess Mg and Sb is less than 0.1%.

Excess Mg is then believed to be a prerequisite to obtain *n*-type Mg_3_Sb_2-*x*_Bi*_x_*. One interesting question arises as to how much excess Mg should be added when synthesizing the polycrystalline samples. Both the defect calculations and single-crystal studies give a simple answer [48, 97]. That is, only slightly excess Mg (less than 0.1%) is necessary to guarantee the system to be *n*-type if efficient dopants are chosen [171, 195, 196]. However, for the experimental preparation of polycrystalline samples, the real conditions are more complicated. As highlighted in the very beginning, Mg is a very reactive element whose surface in the air will soon be covered by MgO. Moreover, in the mixture of raw elements and the high-temperature sintering process, Mg loss can happen and it varies with different milling methods (hand milling or ball milling) and different sintering temperatures and atmospheres. These make it very difficult to accurately control the content of excess Mg in practical experiments. As summarized in Table 3, excess Mg with a range from 0 to even as high as 16% had been used in the initial synthesis by different groups to compensate for the potential loss of Mg. However, since excess Mg is too difficult to enter into the lattice structure to form interstitial Mg defect [48, 97], adding too much excess Mg could result in the Mg-rich phase, as found by Shuai et al. [178]. In another experiment by Imasato et al. [87], they found that too much excess Mg could lead to an increase in the thermal conductivity and thus is detrimental for the overall TE performance. Moreover, too much excess Mg might also affect the stability of the synthesized polycrystalline samples, which will be discussed in the next section. It is worth noting that alloying Bi can lower the temperatures in either the melting or sintering process, which might be helpful to control the loss of Mg.

Under the Mg-excess condition, it is possible to achieve *n*-type Mg_3_Sb_2-*x*_Bi*_x_* by extrinsic doping. The selection of dopants is another key point determining the electrical performance of this TE material. Generally, several aspects need to be considered to find a good dopant: (i) whether a dopant enables the realization of *n*_opt_ since the doping efficiency varies for different elements; (ii) the dopant will induce defects or disorder in the host lattice, which may be detrimental to the carrier mobility. An empirical rule given by Ioffe that was summarized by Wang et al. [197] is as follows: the electron mobility is weakly affected if the dopant is introduced to the sub-lattice that has less contribution to the conduction band [197].

In the historical development of *n*-type Mg_3_Sb_2-*x*_Bi*_x_*, the selection of dopants also follows such empirical criteria. Initially, the anion dopant Te was first selected to realize *n*-type transport [70, 71]. Subsequently, its isoelectronic chalcogen dopants S [86] and Se [84, 93] were also studied, which however are found to be less efficient due to the decreased carrier concentration and mobility [86]. Despite being the most efficient anion dopant, Te doping itself is difficult to realize the *n*_opt_ (up to 1 × 10^20^cm^−3^, Figure 5(e)) in Sb-rich Mg_3_Sb_2-*x*_Bi*_x_*, owing to the limited solubility and doping efficiency (Figure 7(d)). The experimentally obtained maximum Hall carrier concentration is just 2 × 10^19^cm^−3^ through Te doping at anion sites in Mg_3_Sb_2_ [136].

After the significant role of Mg-excess for realizing *n*-type doping was found [97], defect calculations predicted that Mg substitution with trivalent (or higher) cations can be even more effective than Se and Te doping to achieve high electron density above 1 × 10^20^cm^−3^ [171, 195, 196]. In experiments, group 3 elements (Sc and Y) were found to be the most effective cation dopants which not only enable the realization of high carrier concentration (Figure 7(d)) but also have a weak effect on carrier transport [48, 73, 92, 173, 192]. The latter might originate from the smaller differences in the electronegativity and ionic radius between Mg and the dopants [86, 89, 159, 197]. The lanthanide dopants with a larger ionic radius, such as La, Pr, and Ce [115, 118, 193], were also found to be effective in achieving higher electron density than Te doping (Figure 7(d)) buthad an adverse effect on the carrier mobility. Moreover, the cation dopants on the Mg sites seem to have one more advantage than the anion dopants. That is, it can improve the thermal stability as will be discussed in the following section [73, 115].

## 4. TE Device and Thermal Stability

**Figure 8:**
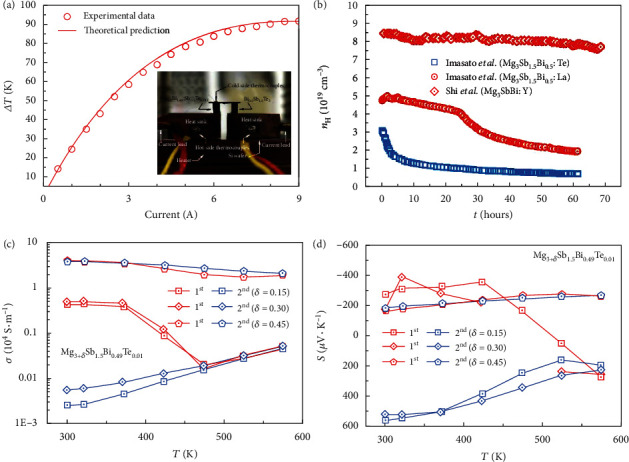
(a) Electrical current dependence of temperature difference (Δ*T*) between the hot and cold sides at the hot-side *T* of 350 K. The inset shows optical image of the experimental setup for the TE cooling measurement with a unicouple consisting of *p*-type Bi_0.5_Sb_1.5_Te_3_ and *n*-type Mg_3.2_Bi_1.498_Sb_0.5_Te_0.002_. (b) Hall carrier concentration versus measurement time for La-doped samples under dynamic vacuum and Y-doped samples under vacuum; the data are taken from Refs. [73, 115]. (c, d) Temperature-dependent electrical conductivity and Seebeck coefficient of Mg_3+*δ*_Sb_1.5_Bi_0.49_Te_0.01_ with different excess Mg in our experimental work. 1^st^ and 2^nd^ denote the first and second heating measurements in the helium atmosphere, respectively. Panel (a) is reproduced with permission from Ref. [33]. Copyright 2019 American Association for the Advancement of Science.

The rapid breakthrough in the TE performance of *n*-type Mg_3_Sb_2-*x*_Bi*_x_* from RT to 700 K ignited the research interest for practical device applications. Mao et al. [33] first reported on a unicouple consisting of *n*-type Mg_3.2_Bi_1.498_Sb_0.5_Te_0.002_ and *p*-type Bi_0.5_Sb_1.5_Te_3_, as shown in the inset of Figure 8(a). When an input electrical current of 9 A is applied and the hot side is maintained at 350 K, the Peltier effect generated by this unicouple gives a maximum temperature difference of 91 K, which is even larger than the commercial Bi_2_Te_3_-based device [33]. This encouraging result suggests the potential of low-cost Mg_3_Sb_2-*x*_Bi*_x_* as a substitute for the current state-of-the-art *n*-type Bi_2_Te_3-*x*_Se*_x_*. However, for practical device applications, there are still important engineering challenges awaiting further studies, including the design principle of the module, electrode fabrication, interface optimization, and protective coating [198–200]. The solutions to these engineering problems rely on the basic chemical and physical properties of Mg_3_Sb_2-*x*_Bi*_x_* alloys. Above all, the stability issues of Mg_3_Sb_2-*x*_Bi*_x_*, including Mg loss and oxidization [65, 177], the decomposition and possible deterioration of TE performance [90, 91] at elevated temperatures, and possible deliquescence under humid environment, need to be well addressed before long-term usage.

The stability problems for Mg_3_Sb_2_ came into attention even in the very early publication by Condron et al. in 2006, in which the studied samples exhibited a *p*-type semiconducting behavior [65]. They found oxygen present at the grain boundaries of the hot-pressed Mg_3_Sb_2_ samples. Mg loss and oxidization were also observed at temperatures above 900 K. In the case of *n*-type Mg_3_Sb_2-*x*_Bi*_x_* alloys, the oxidization of Mg was also observed. Shi et al. have tried to grind the melted Mg_3_Sb_2-*x*_Bi*_x_* ingots into powders under different atmospheres before hot pressing [177]. They found that, if the powders were ground in the glove box with the inert atmosphere, the obtained pellets will show a relatively low level of oxidization. Moreover, Mg deficiency was found at the grain boundary region in the *n*-type Mg_3_Sb_2_ samples by Kuo et al. [98]. In a temperature-dependent powder X-ray diffraction study, Song et al. [91] found that a secondary phase Sb appears even in the first heating process at temperatures above 500 K in both densified bulk samples and powdered samples of *p*-type Mg_2.985_Ag_0.015_Sb_2_. A similar phenomenon also happened to *n*-type Mg_3_Sb_1.475_Bi_0.475_Te_0.05_, in which Jørgensen et al. found an approximately 11 wt.% of elemental bismuth crystallizing as a secondary phase after the first thermal cycle from 300 K to 725 K [90]. In another study by Chen et al. [79], a Bi-rich secondary phase was directly observed using TEM. Furthermore, Mao et al. [33] found that *n*-type Mg_3_Sb_2-*x*_Bi*_x_* in the air will deteriorate if Bi content is increased. This can be understood from the view of chemical bonding. Since the bond length of Mg-Bi is larger than that of Mg-Sb, the diminutive Mg1 atom will more severely deviate the octahedral rule with increasing Bi content in Mg_3_Sb_2-*x*_Bi*_x_* [94], making the interlayer bonding weaker and thus the thermal stability worse.

Luckily, the thermal stability of Mg_3_Sb_2-*x*_Bi*_x_* could be improved if suitable doping elements were chosen. Compared to doping at anion sites, some studies found that both donors and acceptors replacing Mg sites are beneficial for improving the thermal stability, for example, doping Ag for *p*-type Mg_3_Sb_2_ [91] and Y [73], La [115], and Sc [92] for *n*-type Mg_3_Sb_2-*x*_Bi*_x_* (as shown in Figure 8(b)). This might be due to the larger sizes of these doping ions as the structural instability caused by the small size of Mg ions [97] can be improved after cationic doping. The thermal stability could also be improved by increasing nominal Mg content. Song et al. found that the Mg-excess *p*-type Mg_3_Sb_2_ show better stability than Mg-deficient ones [91]. We also checked the role of excess Mg on the electrical performance of Te-doped Mg_3+*δ*_Sb_2-*x*_Bi*_x_* during thermal cycling in the helium atmosphere. As shown in Figures 8(c) and 8(d), the less Mg-excess samples show less stability than the more Mg-excess ones, and the former even show a change of the sign of the Seebeck coefficient during thermal cycling. The excess Mg is demanded to suppress the formation of Mg vacancies enabling *n*-type conduction and also better thermal stability. However, too much excess Mg, as discussed in the above section, will also lead to the oxidization and the increase of thermal conductivity [87], which might be detrimental for the long-term use of Mg_3+*δ*_Sb_2-*x*_Bi*_x_* TE device.

All in all, it can be concluded that Mg plays a crucial role in the stability of this TE system. The small ionic radius of Mg makes it relatively unstable in the lattice of Mg_3_*X*_2_ [97], and Mg loss [70, 192] and the precipitation of the Sb/Bi phase [90, 91] can occur at elevated temperatures owing to the high vapor pressure of Mg. Luckily, doping at the Mg site could stabilize the lattice structure and balance the Mg loss [73, 115]. Considering that these problems are induced by the active Mg, the synthesis of Mg_3+*δ*_Sb_2-*x*_Bi*_x_* materials and the assembly of TE devices need to control strictly the temperature and atmosphere to guarantee stability and reproducibility, particularly for practical applications. These might make the process cost of Mg_3+*δ*_Sb_2-*x*_Bi*_x_* higher than that of Bi_2_Te_3_-based materials, although the cost of the raw elements in the former is lower. Moreover, for practical TE modules, there are additional demands for the exploration of matched *p*-type legs, suitable electrode materials, long-term interface stability, etc. All these aspects need to be comprehensively considered to finally answer the question of whether *n*-type Mg_3+*δ*_Sb_2-*x*_Bi*_x_* can substitute Bi_2_Te_3-*x*_Se*_x_* for low-grade heat power generation and refrigeration.

## 5. Summary and Outlook

The past four years have witnessed the rapid development of *n*-type Mg_3_Sb_2-*x*_Bi*_x_* TE alloys with high *zT* values above 1.5 at 700 K and 0.8 at 300 K. These cheering results make low-cost Mg_3_Sb_2-*x*_Bi*_x_* a promising substitute for state-of-the-art *n*-type Bi_2_Te_3-*x*_Se*_x_* for power generation and refrigeration near RT. The reasons underlying the high TE performance are high band degeneracy and weighted mobility enabling large power factor and strong anharmonicity guaranteeing low lattice thermal conductivity. These good electrical and thermal transport properties are fundamentally determined by the intrinsic crystallographic, electronic, and phononic structures of Mg_3_*X*_2_ strongly linked to the element Mg. The interaction between the Mg1 and Mg2 atoms is a possible origin leading to the high conduction band degeneracy. The diminutive size of Mg, which is too small for the octahedrally coordinated site, leads to weak interlayer bonding and low thermal conductivity but also thermal instability. The close interactions of Mg1-Sb and Mg2-Sb are attributed to the nearly isotropic thermal properties. Moreover, only under the Mg-excess condition can *n*-type doping be realized for the Mg_3_Sb_2-*x*_Bi*_x_* system. However, active Mg also brings problems for both current laboratory research and future applications. That is, Mg loss and oxidation at elevated temperatures could lead to the decomposition of the compounds and deterioration of electrical performance.

With the deep understanding of band structure, the carrier scattering mechanism, and roles of cation dopants, the TE performance of *n*-type Mg_3_Sb_2-*x*_Bi*_x_* is already comparable to that of the state-of-the-art Bi_2_Te_3-*x*_Se*_x_*, laying a good foundation for practical TE applications near RT. Further studies on device design and fabrication are now calling on. In the meantime, the thermal and chemical stabilities of *n*-type Mg_3_Sb_2-*x*_Bi*_x_* alloys, which are strongly related to the active Mg element, will bring real challenges. All the processes, including material synthesis, transfer, joint connection, module assembly, and packaging, might need to be strictly controlled. Suitable interface materials, mechanical property tests, and large-scale production of TE modules are awaiting further studies. Moreover, despite the currently developed unicouple, Mg_3_Sb_2-*x*_Bi*_x_* and Bi_2-*x*_Sb*_x_*Te_3_ as the *n*-type and *p*-type legs, respectively, give a very promising cooling performance, and whether Bi_2-*x*_Sb*_x_*Te_3_ is the best *p*-type material for multipair TE module still needs further studies. The exploration of matched *p*-type material, based on Mg_3_Sb_2-*x*_Bi*_x_* or the other isoelectronic Zintl phase, should be put on the agenda.

## Figures and Tables

**Table 1:** Basic physical and chemical information for the elements in the two *n*-type near-RT Mg_3_Sb_2-*x*_Bi*_x_* and Bi_2_Te_3-*x*_Se*_x_* thermoelectrics. Oxygen is also included since it is often found in the synthesized samples. Most data are obtained from WebElements [101] while Shannon-Prewitt radii from KnowledgeDoor [102].

Element	Mg	Sb	Bi	Te	Se	O
Atomic number	12	51	83	52	34	8
Density (g/cm^3^)	1.738	6.697	9.78	6.24	4.819	—
Abundances (ppm by weight)	29000	0.2	0.025	0.001	0.05	460000
Melting point (°C)	650	630.63	271.3	449.51	221	-218.3
Boiling point (°C)	1090	1387	1564	988	685	-182.9
Electronic configuration	3*s*^2^	5*s*^2^ 5*p*^3^	6*s*^2^ 6*p*^3^	5*s*^2^ 5*p*^4^	4*s*^2^ 4*p*^4^	2*s*^2^ 2*p*^4^
Electronegativity (Pauling)	1.31	2.05	2.02	2.1	2.55	3.44
Atomic radius (Å)	1.5	1.45	1.6	1.4	1.15	0.6
Pauling ionic radius (Å)	0.65 Mg^2+^	—	—	2.21 Te^2-^	1.98 Se^2-^	1.4 O^2-^
Shannon-Prewitt radius (Å) (coordination number)	0.72 Mg^2+^(6)	0.76 Sb^3+^(6)	1.03 Bi^3+^(6)	2.21 Te^2-^(6)	1.98 Se^2-^(6)	1.40 O^2-^(6)

**Table 2:** Lattice parameter and bond length between adjacent atoms for Mg_3_*X*_2_. The data are taken from Refs. [61, 108, 110–112].

Mg_3_Sb_2_			Mg_3_Bi_2_		
Lattice parameter (Å)		Reference	Lattice parameter (Å)		Reference
*a*	*c*		*a*	*c*	
4.573	7.229	[61]	4.671	7.403	[61]
4.568 ± 0.003	7.229 ± 0.004	[108]	4.666	7.401	[110]
4.547(5)	7.235(3)	[111]	4.645(3)	7.380(3)	[111]
Bond length (Å)					
Mg1-Sb(d1)	3.111	[108]	Mg1-Bi(d1)	3.1403	[112]
Mg2-Sb(d2)	2.819		Mg2-Bi(d2)	2.9132	
Mg2-Sb(d3)	2.933		Mg2-Bi(d3)	2.9900	
Mg1-Mg2(d4)	3.736		Mg1-Mg2(d4)	—	

**Table 3:** The nominal element contents used by different groups to synthesize Mg_3_Sb_2-*x*_Bi*_x_* alloys. The preparation method, Hall carrier concentration, and peak *zT* are also shown. The data are taken from Refs. [33, 47, 48, 70–73, 80, 82, 84–88, 92, 93, 115, 192, 194].

Nominal content			Method	*n*_H_ (10^19^ cm^−3^)	*zT*	Reference
Mg	Bi	Dopant				
3.2	0.49	Te	BM+HP (Ar)	2	1.5@700 K	[70]
		0.01				
3	0.48	Te	AM+BM+SPS (Vac.)	2.2	1.65@725 K	[71]
		0.04				
3.07	0.48	Se	AM+BM+SPS (Vac.)	0.91	1.23@725 K	[84]
		0.02				
3.1	0.49	TM&Te	BM+HP	3-4.4	1.5-1.7@773 K	[72, 85]
		0.1&0.01				
3.2	0.49	Te	BM+HP	2.0-3.5	1.4-1.5@773 K	[192]
		0.01				
3	0.49	S	AM+BM+SPS (Vac.)	0.576	1.0@725 K	[86]
		0.01				
3.02	0.49-0.89	Mn&Te	BM+SPS	\	1.5-1.6@773 K	[80]
		0.01&0.01				
3.01-3.2	0.49	Te	BM+HP (Ar)	2.8-3.5	1.2-1.4@600 K	[87]
		0.01				
3.05	0.5	La	BM+HP (Ar)	5	1.0@600 K	[114]
		0.005				
3.2	0.49	Se	BM+HP	1.9	1.4@723 K	[93]
		0.01				
3.15	0.49	Mn&Se	BM+HP	2.1	1.7@623 K	[93]
		0.05&0.01				
3.032	1	Y	Melting+cut+HP	7.1	1.8@700 K	[73]
		0.018				
3.045	1	Sc	Melting+cut+HP	3.4	1.3@500 K	[92]
		0.005				
3.05	1.39825-1.393	Te	BM+HP (Ar)	2.3-6	1.0-1.2@400-500 K	[47]
		0.01				
3.02	0.5	Y	BM+HP	3.6	1.8@773 K	[88]
		0.02				
3.2	1.298-1.498	Te	BM+HP	1.4	0.9@350 K	[33]
		0.002				
3.5	0	Sc&Te	Powder mix+SPS	2	1.5@725 K	[190]
		0.03&0.04				
3.01	0.49	Te	BM+HP (Ar)+Mg-vapor annealing	2.8	0.8@300 K, 1.4@700 K	[82]
		0.01				
>3	1.25	Y	Mg-flux	1.6	0.82@315 K	[48]
		\				

BM: ball milling; HP: hot pressing; AM: arc melting; SPS: spark plasma sintering; Vac.: vacuum; TM: transition metal.
